# A Journey into Atopic Dermatitis: 5 Experts’ Reviews Appearing in DPCJ Next Issues

**DOI:** 10.5826/dpc.1104a147

**Published:** 2021-10-01

**Authors:** Anna Balato

**Affiliations:** 1 Unit of Dermatology – University of Campania “Luigi Vanvitelli”, Naples, Italy

In the last decade, our understanding of Atopic Dermatitis (AD) pathogenesis made significant steps forward leading to the development of multiple game-changer therapies. Although we can say that the horizon is now a little brighter, we cannot argue that “all the job” is done. We are currently witnessing a translational revolution in the treatment and management of AD. Breakthroughs in the understanding of the immunology and the genetics of AD have been translated into highly effective targeted therapies. Unraveling the importance of the IL-4 and IL-13/Th2 immune response and associated signaling pathways have directly led to the development of some of the most effective treatments of AD to date.

The aim of this “journey” is to provide further insights into AD, collecting dedicated article reviews by experts in the field.

It is now well established that immunological response has a leading role in the pathogenesis of AD, so “Lessons from Immunology” will be one of the steps in this journey. Over the last years, dermatology has greatly expanded its field of research, going beyond the focus on skin, with an eye winked at immunology research. In this specific review, the scientific pathogenetic rationale that stands beneath the mechanisms of action of modern drugs will be addressed. Even if it is true that dermatology has gone through a radical transformation in its approach, it is fundamental to mention that dermatology was born as a morphologic science, and as such, the clinical approach could not miss. In particular, all clinical manifestations and attempts to harmonize clinical criteria for AD classification will be discussed in the review on “Epidemiology and Clinical Phenotypes”.

In this translational revolution process, we could not help but to consider updating on “The New Era of Biologics in AD” and on the “JAK Inhibitors in the Treatment of AD”. Anyhow, we asked ourselves a question: “Have Topical and Conventional Systemic Treatments in AD Gone Out of Fashion?”.

These important themes will be discussed in separate reviews in order to provide a very recent update to help to better understand this disease and improve the management of AD patients.

Enjoy the reading!

